# Improving Sustainability of the Griess Reaction by Reagent Stabilization on PDMS Membranes and ZnNPs as Reductor of Nitrates: Application to Different Water Samples

**DOI:** 10.3390/polym14030464

**Published:** 2022-01-24

**Authors:** Lusine Hakobyan, Belén Monforte-Gómez, Yolanda Moliner-Martínez, Carmen Molins-Legua, Pilar Campíns-Falcó

**Affiliations:** MINTOTA Research Group, Departament de Química Analítica, Facultat de Química, Universitat de València, Dr. Moliner 50, 46100 Valencia, Spain; lusine.hakobyan@uv.es (L.H.); mongobe@alumni.uv.es (B.M.-G.); yolanda.moliner@uv.es (Y.M.-M.)

**Keywords:** PDMS membranes, embedded Griess reagent, ionic liquid, ZnNPs, nitrate and nitrite, smartphone, digital imagen, colorimetry, real samples, 96 wells

## Abstract

A new approach based on the use of polydimethylsiloxane (PDMS) membranes doped with Griess reagents for in situ determination of ${\mathrm{NO}}_{2}^{-}$ and ${\mathrm{NO}}_{3}^{-}$^-^ in real samples is proposed. The influence of some doping compounds, on the properties of the PDMS membranes, such as tetraethyl orthosilicate (TEOS), or/and ionic liquids (OMIM PF_6_) has been studied. Membrane characterization was performed. To apply the procedure to ${\mathrm{NO}}_{3}^{-}$ determination, dispersed Zn nanoparticles (ZnNPs) were employed. The analytical responses were the absorbance or the RGB components from digital images. Good precision (RSD < 8%) and detection limit of 0.01 and 0.5 mgL^−1^ for ${\mathrm{NO}}_{2}^{-}$ and ${\mathrm{NO}}_{3}^{-}$, respectively, were achieved. The approach was satisfactory when applied to the determination of ${\mathrm{NO}}_{2}^{-}$ and ${\mathrm{NO}}_{3}^{-}$ in drinking waters, irrigation and river waters, and waters from canned and fresh vegetables. The results obtained were statistically comparable with those by using nitrate ISE or UV measurement. This approach was transferred satisfactory to 96 wells for multianalysis. This study enables the improvement in the on-site determination of ${\mathrm{NO}}_{2}^{-}$ and ${\mathrm{NO}}_{3}^{-}$ in several matrices. It is a sustainable alternative over the reagent derivatizations in solution and presents several advantages such as being versatile, simplicity, low analysis time, cost, and energy efficiency. The response can be detected visually or by portable instruments such as smartphone.

## 1. Introduction

Nitrite and nitrate are widely present in environmental samples like water, soil, food and agricultural products. Elevated concentrations of nitrate in water systems pose a significant risk to the environment and to human health [1]. The quantity of nitrates in waters and soil contribute to the amount in vegetables and fruits. Vegetables are the most important source of nitrate exposure in the human diet and contribute to the intake of more than 80% of nitrates [2]. In Table 1 are summarized the main regulations about nitrate concentration in water and in some foods (vegetables) [3,4,5,6,7,8]. The maximum nitrate amount to be ingested daily is less than 3.65 mg kg^−1^ by body weight [9]. Because of this concern, during the past 15 years, numerous methods have been reported for the detection and determination of nitrite and/or nitrate including spectrophotometric, chemiluminescent, electrochemical, chromatographic, capillary electrophoresis, spectrofluorometric and electrochemiluminescence methods [10]. Moreover, several reviews have been published [10,11]. Among these methods, the spectroscopic methods have excellent detection limits and have facile protocols. These methods are by far the most widely used due to its simplicity and cheapness. The well-known spectrophotometric method for analysis of nitrite is based on the Griess reaction [12]. Often the protocols described for this determination indicate that the procedure should be carried out in the laboratory either in batch or continuous mode (flow injection analysis). These methodologies are far from the actual needs of analytical methods that combine high sensitivity, accuracy and rapid analysis with simplicity, portability, low cost and access for non-qualified citizen groups. Thus, to develop in situ procedures, the Griess reaction presents some weak points, such as (i) the reagents being added in solution and (ii) the determination of nitrate required its reduction to nitrite. Concerning to Griess reagents, these are rather unstable and usually need to be keeping at low temperatures as individual solutions. One option to stabilize reagents is to embed them in solid supports; materials such as polymers can be used as an inert matrix support [13,14]. This approach is being used for the development of optical sensors and microfluidic devices [15,16,17,18]. These strategies generally allow the miniaturization, reduce reagent and waste, cost, not requiring any external forces, and they can be used for in situ analysis by non-trained personal. Moreover, the reagent entrapment during the polymeric gelation process has certain advantages such as greater resistance of the membrane or a better preservation of the reagent against environmental conditions. Campíns-Falco et al. [16,17,18] have entrapped reagents such as 1,2-Naftoquinone sulphonate (NQS) or tetramethylbencidine (TMB) in PDMS matrix with satisfactory results. Bhakta S.A. et al. proposed a paper colorimetric test for ${\mathrm{NO}}_{2}^{-}$ using paper and for which several alternatives have been studied to avoid the deterioration of the reagent [19]. We selected PDMS as and hydrofobic material to protect the reagent. The reagents keep all the properties when PDMS was used as supporting material. These membranes can be doped with ionic liquid to enhance the permeability of gaseous analytes (ammonia) [20]. Doped polymeric reactions can be used for catalytic reactions, this is the case of polysulfone membranes doped with ionic liquid [21], or polybenzimidazole-based nanofiltration membrane doped with azido derivatized cinchona-squaramide bifunctional catalyst [22].

Concerning to the nitrate reduction to nitrite, enzymatic reduction using nitrate reductase or photochemical reduction through the use of UV light can be used; however, these methods typically offer poor reproducibility [23]. Other studies have focused on the nitrate reduction approaches using a column of Zn granules or a copper-coated cadmium column [24,25] hydrazide with copper catalyst [26] photo-induced device, etc. Vanadium III chloride as reductant has also been proposed [27,28]. Many of these procedures are performed on-line by using flow injection analysis (FIA) to control the reduction process and the derivatization step. In reference to in situ procedures Martínez-Cisneros et al. [29] proposed a lab on a chip procedure using a column of cadmium and Griess reaction. M. Jayawardane et al. [30] used immobilized Zn dust for the reduction reaction and developed a microfluidic paper-based procedure. In recent years, the use of nanoparticles has aroused great interest for their especial properties. As far as we know no work has been published using NPs as reductor of nitrates combined with Griess reaction.

In the present work we present an in situ, low-cost, robust sensor based on Griess reagents entrapped in a polymeric composite as a delivery reagent support (membrane). Due to the higher porosity, PDMS doped with liquid ionic has been selected as a composite. Additionally, the reduction capacity of non-toxic ZnNPs on the reduction reaction of nitrate has been studied. The optimized procedure has been successfully applied to determine nitrite and nitrate in several real samples (water from different sources, and water from canned vegetables and boiled fresh vegetables). A comparison with other methods reported in the literature has been performed. This method simplifies the analytical protocol and reduces the toxicity and manipulation of solutions. This is a fast procedure that can be used as a single assay or in a multiple assay by using microplate (e.g., 96 wells) with reagent-PDMS-membranes settled at the bottom of the wells. The analytical responses were obtained by measuring the absorbance or by using the RGB components from digital images obtained with a smartphone.

## 2. Materials and Methods

### 2.1. Reagents and Solutions

Ultrapure water obtained using a Nanopure II system (Barnstead, NH, USA) was employed for the preparation and dilution of all the solutions. PDMS membranes were synthesized by using Sylgard^®^ 184 Silicone Elastomer Kit (base and curing agent) obtained by Dow Corning (Midland, MI, USA). Tetraethyl orthosilicate (TEOS ≥ 99.0%), silicon dioxide nanoparticles (SiO_2_NPs, 99.5%, 5–15 nm particle size), N-(1-Naphthyl) ethylenediamine dihydrochloride (NEDD), 1-methyl-3-octylimidazolium hexafluorophosphate (OMIM PF_6_), Zinc nanopowder (ZnNPs, 99%, 40–60 nm particle size), hexadecyltrimethylammonium bromide (CTAB) were provided by Sigma-Aldrich (St. Louis, MO, USA). Sulfanilamide (SA) was obtained from Guinama (Valencia, Spain). Sodium Dodecyl Sulphate (SDS), potassium nitrate and anhydrous citric acid were provided by Panreac (Barcelona, Spain). Zn powder and sodium nitrite were purchased from Probus (Badalona, Spain). Silver nitrate was obtained from Scharlab (Barcelona, Spain).

### 2.2. Apparatus

Absorbance measurements were carried out using a Cary 60 UV–vis spectrophotometer. Spectra were recorded from 200 to 1000 cm^−1^. For data collection and processing, CaryWinUV software was used (Agilent Technologies, Santa Clara, CA, USA). For preparing the ZnNPs dispersion and PDMS sensors devise was employed ultrasonic bath from Sonitech. Size distributions of the Zn nanoparticles were determined with a Malvern Zetasizer Nano ZS from Malvern Panalytical Ltd. (Malvern, UK). A pH-meter Crison micro pH 2001, (Crison Instruments S.A., Barcelona, Spain) and a nitrate electrode were used for potentiometric measurement of nitrate. Morphology of the membrane was studied with a Hitachi S-4800 scanning electron microscope at an accelerating voltage of 20 keV, over metalized samples with a mixture of gold and palladium for 30 s. The LG Optimus L5 II smartphone (LG, Seoul, Korea) was used to take photos of the solutions. The images were analyzed by the open-source software ImageJ. This software was employed to evaluate the color intensity of the pictures. In this model the maximum values of all the channels give rise to the white color, while if all the values are zero, black color is obtained. The quantification of the green component was chosen because it provided the best results. The color intensity obtained was converted into a value of absorbance through the expression A = −log B/255, where B is the value of the coordinate and 255 represents the maximal transmitted light intensity.

### 2.3. Preparation of the Composites

#### 2.3.1. PDMS/TEOS-SiO_2_NPs-SA-NEDD

The fabrication of the PDMS/TEOS-SiO_2_NPs-SA-NEDD composites was carried out following the procedure proposed in [13] with some modifications. Firstly, the reagents sulphanilamide (SA) (4.18%) and N-1-Naphthyl ethylenediamine dihydrochloride (NEDD) (1.14%) were added to the TEOS (39.77%) and SiO_2_NPs (0.11%) dispersion previously prepared. The percentages of the different components were in weight. To achieve homogeneity, an ultrasonic bath was used for 5 min. After, the final mixture was added to the elastomer base (49.82%) and the new mixture was vigorously stirred for 15 min at room temperature to obtain a homogeneous suspension. Subsequently, the curing agent (4.98%) was added to the previous solution leaving 5 min under stirring. The standard mixing ratio for PDMS was 10:1 elastomer and curing agent, respectively. This ratio provides the desirable and optimum mechanical properties. The gelation procedure was carried out at 30 °C for 8 h, depositing 200 μL of the final mixture in the well-plates (d = 1.5 cm). In case of using microplate wells, 20 ul were dropped in each well.

#### 2.3.2. PDMS/TEOS-SiO_2_NPs-SA-NEDD-OMIM PF_6_

The preparation of the supported IL-based device was performed by mixing the reagents that form the azo compound, such as SA (4.18%) and NEDD (1.14%), with the OMIM PF_6_ (7%). The mixture was stirred for 10 min. After, the elastomer base (44.2%) was added to the previous mixture and the resulting combination (combination 1) was stirred during 10 min more to get a homogeneous dispersion. Then, a mixture of TEOS-SiO_2_NPs was prepared by mixing SiO_2_NPs (0.11%) with TEOS (39.77%) (combination 2). Finally, combination 1 and 2 were mixed and stirred vigorously to obtain homogenous mixture. After adding the curing agent (4.4), the mixture was stirred for 5 min. Finally, the drying procedure was carried out as previously described (Section 2.3.1).

#### 2.3.3. PDMS/SiO_2_NPs-SA-NEDD-OMIM PF_6_

The procedure followed was like that described in Section 2.3.2, however, in this case the TEOS was not included in the membrane. The proportion of the elastomer base was 84.04%.

#### 2.3.4. Preparation of ZnNPs Dispersion

Solutions of surfactants in water were prepared by weighing the appropriate amounts of CTAB and SDS for final concentrations 15 mM and 17.3 mM, respectively. Mixtures of CTAB and SDS were prepared by mixing the proportions CTAB70% -SDS30% and vice versa. The solutions and mixtures of surfactants (50 mL) were added to 30 mg of ZnNPs, sonicating for 15 min which led to the formation of dispersions of ZnNPs in surfactants. The percentage of the different components in the final dispersion were (45:50:5, SDS:CTAB:ZnNPs). The suspensions were then aged overnight at room temperature. The appropriate amount of dispersed ZnNPs solution was passed through a nylon filter (1 cm diameter) and the ZnNPs were adsorbed.

### 2.4. Analytical Response Measurements

#### 2.4.1. Determination of Nitrites or Nitrates Adding the Griess Reagents in Solution

The determination of nitrites in the solution was carried out by adding 0.5 mL of Griess reagent solution and 0.5 mL of nitrite solution. After 8 min, the absorbance at 540 nm was measured. To determinate nitrates, prior to the Griess reaction, the appropriate volume of dispersed ZnNPs solution as a reducing agent was added (leaving 3 min).

#### 2.4.2. Analytical Response Measurements Adding the Sensor Membrane

The measurement of nitrites by using the synthesized composite was performed by introducing the PDMS-membranes in a vial containing 0.5 mL of citric acid (330 mM) and adding the 0.5 mL of standard solution of ${\mathrm{NO}}_{2}^{-}$. To determinate nitrates, prior to the Griess reaction, the appropriate volume of dispersed ZnNPs or nylon membrane with ZnNPs as a reducing agent was added. The smartphone images were captured and processed [31].

#### 2.4.3. Reference Methods: Potentiometric Measurement and UV at 220 nm

Potentiometric method based on the employment of ${\mathrm{NO}}_{3}^{-}$ ISE electrode was used [28]. In water vegetable samples a saturated solution of AgNO_3_ was added to avoid the presence of Cl^−^ in the samples (vegetable samples). Any sample treatment was performed to determine ${\mathrm{NO}}_{3}^{-}$ in water samples being the spectrophotometric UV method the method used as reference [32].

#### 2.4.4. Analytical Response Measurements in 96-Well Multiplate

In this case a microplate of 96 wells was used. Firstly, the reduction reaction was carried out and the dispersion or the nylon with ZnNPs were place in the well. The composite was place at the bottom of the well and 150 µL of citrate buffer (330 mM) and 150 µL of standard or sample were added.

### 2.5. Analysis of Real Samples

Different types of water samples were analyzed (drinking and tap water and waters from different places of Valencia Community and from irrigation canals) and water from canned and boiled fresh vegetables (spinach and chard).

Wells water, well diluted in proportion 1:3, and irrigation canal water were directly analyzed. Drinking and tap water were processed directly without any previous treatment while river and lake samples were diluted depending on their concentration. The lake sample was diluted to 100 mL and river water—1 mL diluted to 10 mL. If the samples contained particles, they were first filtered. Canned chard and spinach were purchased from the supermarket. Afterwards, 1 mL of the liquid solution was taken and diluted to 100 mL with nanopure water. The diluted solution was used for further analysis. Fresh spinach and chard were cleaned with water and cut in pieces. For the experiments, 140 g of fresh spinach or 280 g of fresh chard were boiled in 350 mL of nanopure water for 20 min. The vegetables were drained and separated from the liquid. The resulting liquid was diluted to 250 mL with nanopure water. Then 1 mL of the diluted solution was taken and diluted to 100 mL. These dilutions were used for further analysis.

## 3. Results and Discussion

### 3.1. Study of the Reaction with the Composite

Our research group has experience in the development of polymeric composites in which derivatizing reagents can be embedded and stabilized in time. In this paper, both reagents involved in Griess reaction were entrapped in PDMS membranes. Taking into account the ratio and the number of reagents used in solution, these reagents were entrapped in a PDMS membrane. In the first set of experiments, the response of PDMS sensors to the ${\mathrm{NO}}_{2}^{-}$ concentration was evaluated. It was observed that when a sensor was introduced into a solution, the reagents (SA and NEDD) were released from the membrane to the solution. No differences in the analytical signals were obtained by entrapping both reagents (SA and NEDD) in the same PDMS composite or in different. Thus, the entrapment in the same membrane was the option selected.

**Figure 1:**
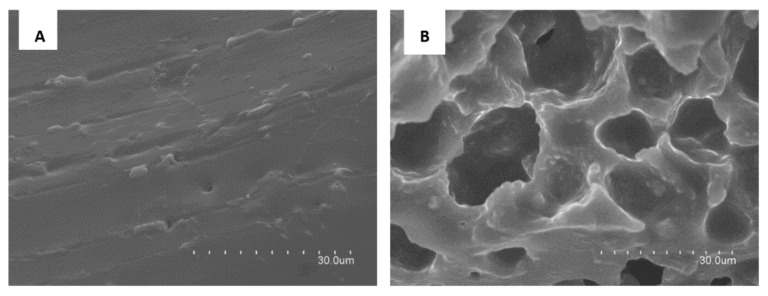
SEM imagens corresponding to the PDMS-TEOS (**A**) and PDMS-OMIM PF_6_ (**B**) membranes.

To obtain similar sensitivity to that obtained by performing this reaction in solution, the composition of the membrane was studied. One of the possible drawbacks of PDMS membranes can be the low reagent diffusion; therefore, with the purpose of increasing diffusion, the membranes were doped with TEOS and/or ionic liquid (IL). The addition of TEOS to the membrane improves the hydrophilic character of the membrane [13]. ILs as chemical additives influenced the sol–gel porosity due to the interactions between the components of the polymeric matrix. Based on previous studies realized by Campins et al. [20], the IL selected was 1-Methyl-3-octylimidazolium hexafluorophosphate (OMIM PF_6_), which is hydrophobic and water-insoluble. This compound was confined into the organic polymeric membrane. The cross-linking sol–gel reaction was initiated upon stirring the mixture and the IL was entrapped as a solid, forming a sponge-like PDMS membrane. Simultaneous H-bonds between PF_6_ and silica matrix together with imidazolium groups π-π stacking [33] when using OMIM PF_6_ provide a high-porosity membrane, which can improve the efficiency of the material for sensing purposes. According to Sasikumar et al. [34], the IL are entrapped in the tight spaces between individual polymer chains or clusters. Physical and chemical interactions between the polymer and OMIM PF_6_ can stabilize the ILs in the polymer matrix [35]. The amount of OMIM PF_6_ added to the PDMS was lower than 7% that was the maximum amount allowed to gel properly [20]. Figure 1 shows the SEM image corresponding to the PDMS-TEOS and PDMS-OMIM PF_6_ composites. In presence of OMIM PF_6_ the SEM image shows the spongelike PDMS structures. Similar results have been achieved by A.I. Horowitz and M.J. Panzer [36] who obtained an asymmetric structure of PDMS polymer with high permeability caused by a decreasing rigidity of the polymer backbone and an increase in void volume available for the diffusion of the permeable molecules. The capabilities of the different synthesized membranes (PDMS/TEOS-SiO_2_NPs-SA-NEDD, PDMS/TEOS-SiO_2_NPs-SA-NEDD-OMIM PF_6_ and PDMS/SiO_2_NPs-SA-NEDD-OMIM PF_6_) in terms of reagent release were evaluated and compared.

**Figure 2:**
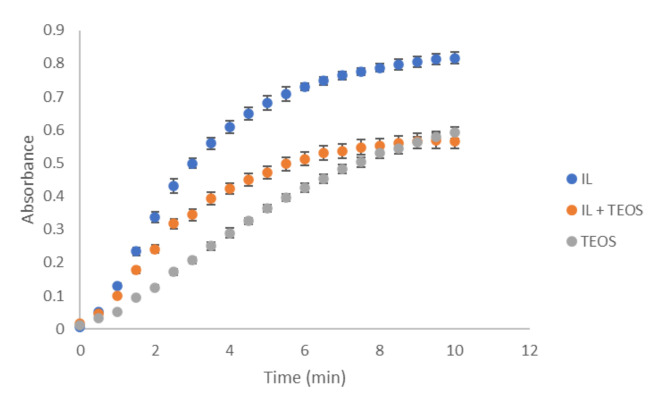
Kinetic study—analytical signals of derivates in solution by using the different type of sensor. ${\mathrm{NO}}_{2}^{-}$. concentration 1.4 mg L^−1^.

The kinetics between ${\mathrm{NO}}_{2}^{-}$ and Griess reagent can be seen in Figure 2. The analytical signal depends on the release of the reagent (SA-NEDD) which in turn depends on the composition of the membrane. The results showed that the analytical responses were higher with the OMIM-PF_6_ modified PDMS membrane than with PDMS-TEOS membrane due to the higher reagent diffusion. The use of OMIM PF_6_ improved sensibility and response time owing to increase the accessibility and the porosity of the membrane. Thus, based on these results PDMS-OMIM PF_6_ with both reagents (SA and NEDD) entrapped in the same support were used for further experiments.

**Figure 3:**
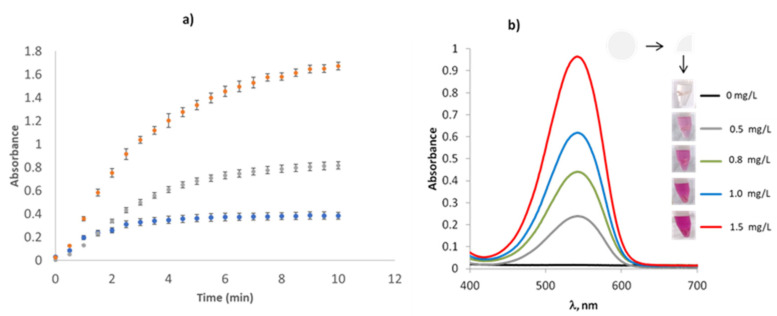
(**a**) Griess reaction time optimization employed PDMS sensing devices for different nitrite concentrations. (**b**) Vis spectra of different ${\mathrm{NO}}_{2}^{-}$ solutions employing Griess reagents entrapped in PDMS membranes.

By using this composite, a kinetic study was carried out in order to determine the time required to reach a plateau. As can be seen in Figure 3a, the analytical signal was dependent on nitrite concentration and in all cases a plate was reached at 8 min. According to these results, this time was selected for further experiments. As illustrative example, in Figure 3b are shown the spectra obtained for different nitrite solutions, as well as a picture of the corresponding solutions in presence of the composite. No significant differences were observed between membranes prepared in different batches. 

The IR spectra were used to characterize the selected membrane. The observed vibrational wavenumbers of the PDMS/SiO_2_NPs-SA-NEDD-OMIM PF_6_ sensor were compared with the PDMS-IL membrane and with the characteristic bands of Griess reagent. As we can see in Figure S1 (red line) several characteristic PDMS bands (2962 cm^−1^, 1256 cm^−1^) were present in the composition of the PDMS/SiO_2_NPs-SA-NEDD-OMIM PF_6_ sensor [37]. The characteristic bands of SA and NEDD were also identified in the proposed sensor. SA were characterized with two bands at 3478 and 3375 cm^−1^ due to the asymmetric and symmetric stretching vibrations of the NH_2_ group and one band at 3267 cm^−1^ due to the symmetric stretching corresponding to the SO_2_NH_2_ group [38]. It was also the characteristic bands between 1600–1400 cm^−1^ related to N-H (d)vibrations. It is possible to see the band around 1300 cm^−1^ corresponding to S=O symmetric. In relation to NEDD, the bands at 3478 and 3375 cm^−1^ can be glimpsed in a very weak form. Characteristic bands within the range 1600–1400 cm^−1^ were identified related to stretching C=C and bending vibration of N-H [39]. (Supplementary Material Figure S1). Membranes were also characterized by EDX in order to find the elemental composition. In PDMS membranes, C, O, and Si were the majority component. The addition of OMIM PF_6_ gave a F in the composition and when the SA and NED were present in the membrane, beside the components already indicated, appeared a band corresponding to N (Figure S2).

### 3.2. Study of ZnNPs as Reductor Nitrate Agent

Some procedures have been described in the bibliography to reduce nitrate to nitrite using Zn powder either in batch mode [40] or in FIA mode using a Zn column [24]. In order to perform batch procedures, the nitrate reduction reagents have to be added to the solution. The insolubility of Zn powder in water solution [41] affects the nitrate reduction reaction, and the reproducibility of the results. Thus, in this paper we propose the use of ZnNPs in order to improve the reduction reaction. One of the limitations of ZnNPs is the low stability of aqueous dispersions. Usually, they present agglomeration which is caused by Van der Waals attraction forces between particles. To disperse NPs in aqueous medium, an external force is needed to overcome the van der Waals attractions and sonication is commonly used to break up agglomerated NPs [42]. In this case, the size of commercial NPs ranged between 40–60 nm, the primary particles were forming agglomerates of micrometers. Sonication was not enough to disperse ZnNPs in water, and surfactants were used.

**Figure 4:**
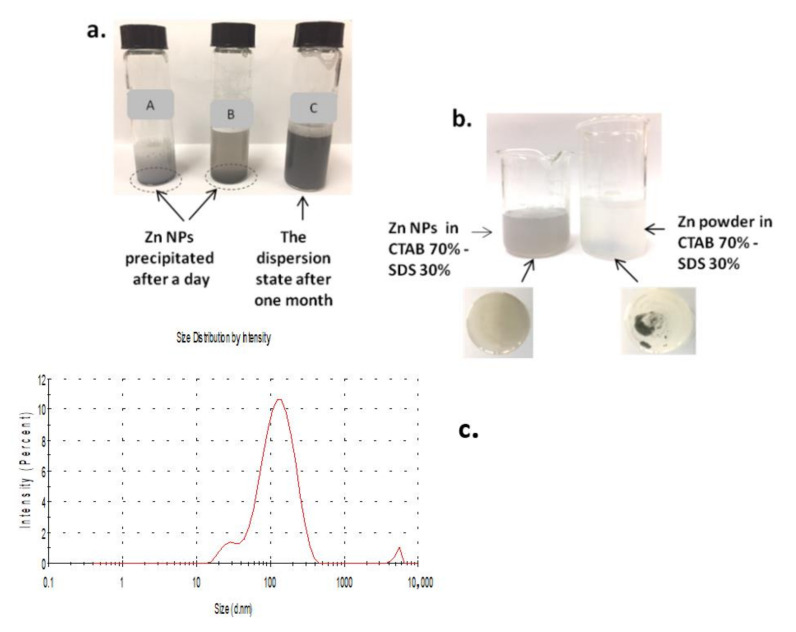
(**a**) ZnNPs in different proportions of surfactants A—CTAB 30%–SDS 70%; B—SDS 100%; C—CTAB 70%–SDS 30%. (**b**) ZnNPs and Zn power in CTAB–SDS surfactant mixture. (**c**) Intensity size distribution of the ZnNPs dispersed in the 30%SDS–70%CTAB surfactant mixture.

Cationic (hexadecyltrimethylammonium bromide-CATB) and anionic surfactants (sodium dodecyl sulphate-SDS) were employed in order to obtain a dispersion of ZnNPs. The solutions of surfactants (CTAB and SDS) are transparent, but in conditions of mixtures with high concentrations produce turbidity, and even precipitation. The non-transparent solutions contained vesicles that are generally stable for a long period of time [43]. According to Tomasic et al. [44] for equal concentrations (25 mM) of both surfactants (CTAB and SDS), precipitate was formed; while for 25 mM CTAB, and 15 mM SDS and vice versa, the vesicles were formed. Based on the above data, mixtures with concentrations lower than 25 mM for CTAB and SDS were prepared. Aqueous solutions with concentrations (15 mM CATB and 17.3 mM SDS) and mixtures in different proportions (0: 100, 30:70, 70:30, 100: 0) of SDS:CTAB respectively were prepared. The ZnNPs were dispersed in all these solutions except in solution 100% of SDS or CTAB. SDS 30%–CTAB 70% was chosen as the final composition for their capacity of forming vesicles (Figure S3A) and its greater stability (more than 1 week) (Figure 4a–c). The presence of these structures allowed stable dispersion (Figure S3B). It should be noted that dispersion of Zn powder under the same conditions was not achieved (Figure 4b). The size of the ZnNPs in the 30% SDS–70%CTAB dispersion was checked by measuring the size distribution using Dinamic Light Scattering (DLS). As shown in Figure 4c, the hydrodynamic size of ZnNPs was around 100 nm by using (DLS) and ζ-potential was 15 mV.

**Figure 5:**
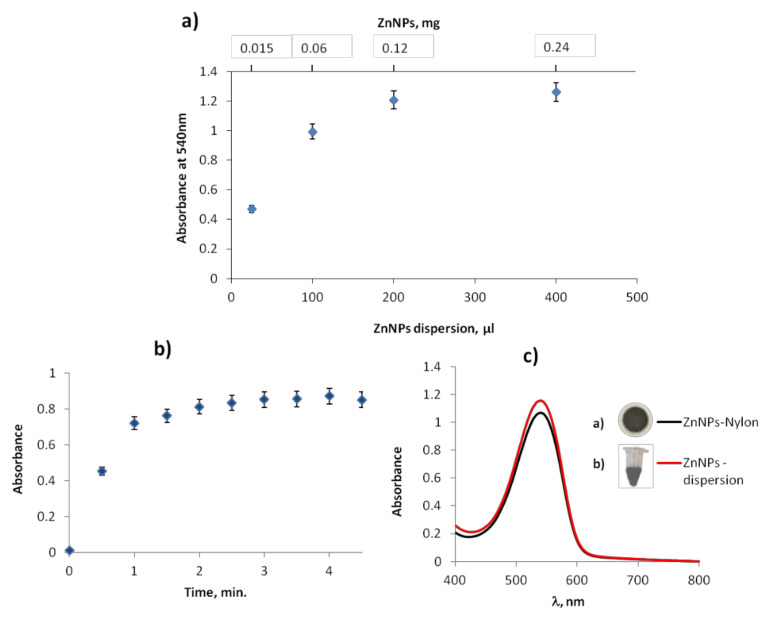
(**a**) ZnNPs amount optimization in the reduction reaction of nitrate (24 mg L^−1^) to nitrite. (**b**) Reduction reaction time optimization of nitrate (20 mg L^−1^) to nitrite. (**c**) Analytical responses using ZnNPs-nylon and ZnNPs-dispersion.

The effect of the amount of ZnNPs in the reduction reaction and in the Griess reaction was evaluated. In order to obtain the satisfactory analytical response, ZnNPs amount required to perform the reduction reaction was studied. As can be seen in Figure 5a, 0.12 mg/per assay (or 200 µL of ZnNPs dispersion) were needed to reach the plateau. Remarkably, this amount was lower than that needed when NPs were not dispersed (0.625 mg). Concerning the time required for the reduction reaction, in Figure 5b is shown the kinetic graph. The results indicated that the reaction was completed within 2 min, and the absorbance did not change over time. No interferences were observed by the presence of the ZnNPs dispersion on the Griess reaction. These results indicated that the dispersed ZnNPs favored the reaction and reduced the amount and time required. To perform the reduction, in this approach ZnNPs dispersion (200 µL) were added to the standard ${\mathrm{NO}}_{3}^{-}$ solutions or samples. After 3 min, the reagent membrane was added to the solution and the total time to carry out both reactions was 10 min. The reaction product (azo compound formation) was stable over time. 

Additionally, a more advance strategy relies on the immobilization of ZnNPs on a nylon membrane (0.45 µm porous size). To this end, 200 µL of a ZnNPs surfactant dispersion were passed through the nylon membrane. The vesicles ad the ZnNPs were retained on the membranes. This membrane was used for reducing ${\mathrm{NO}}_{3}^{-}$. The analytical responses were slightly lower than those obtained with ZnNPs dispersion in surfactant (Figure 5c). However, the stability of ZnNPs was improved compared with liquid dispersion (more than 1 month) than in the dispersion. In this approach, both membranes were added at the same time, and acidic medium was required to release ZnNPs from the nylon membrane to the solution.

### 3.3. Transferability to 96 Wells in a Microplate

In order to develop a method in multiplex format, this approach was transferred to a 96 wells microplate. The size of the membrane used was reduced according to the volume used. In this case, the absorbance could be measured by a microplate reader or by RGB coordinates obtained from the digital images captured by using a smartphone [17,31].

**Figure 6:**
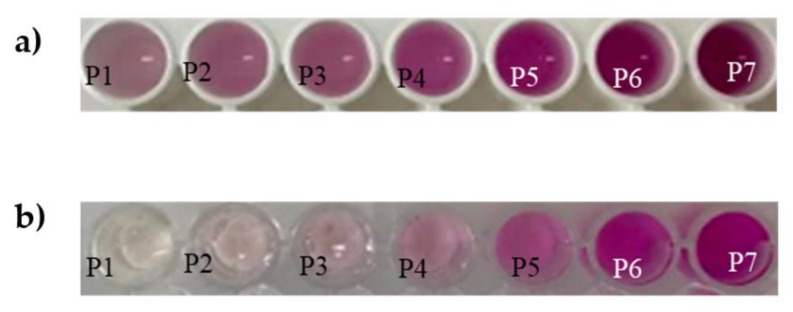
Images corresponding to different concentrations of ${\mathrm{NO}}_{2}^{-}$ (from 0.01 mg L^−1^ to 2.7 mg L^−1^) in 96-microplate of ${\mathrm{NO}}_{2}^{-}$. (**a**) white microplate; (**b**) transparent microplate.

**Figure 7:**
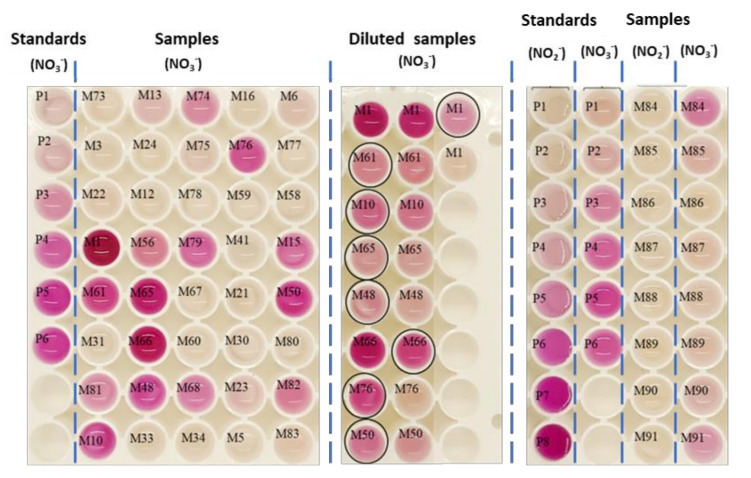
Calibration curve in 96-microplate of ${\mathrm{NO}}_{2}^{-}$ (from 0.01 mg L^−1^ to 2.7 mg L^−1^) (P). ${\mathrm{NO}}_{3}^{-}$ (1.6 to 25 mg L^−1^) (P). Response to ${\mathrm{NO}}_{2}^{-}$ and ${\mathrm{NO}}_{3}^{-}$ in different real samples (M).

To determine ${\mathrm{NO}}_{2}^{-}$, the membrane was fitted at the bottom of the plate and then the citric acid and the sample were added. Two different formats of microplate were tested, transparent and white (Figure 6). The best results were obtained with white plates, and the RGB selected was G (Green) due to the improvement of sensibility and linearity. The experiments were performed by using dairy light or LEDs source (white box), and better results were obtained by controlling the light conditions, in this case by using LEDs. Concerning to the reduction of ${\mathrm{NO}}_{3}^{-}$ to ${\mathrm{NO}}_{2}^{-}$ both methodologies (addition of the ZnNPs dispersion or addition of nylon with ZnNPs retained) provided good results. Figure 7 shows a calibration curve of both analytes, ${\mathrm{NO}}_{3}^{-}$ and ${\mathrm{NO}}_{2}^{-}$, under the optimal conditions. The response was obtained at 1 min) for ${\mathrm{NO}}_{2}^{-}$, and 10 min for ${\mathrm{NO}}_{3}^{-}$, respectively. These responses were stable for at least 120 min, and the presence of ZnNPs did not affect the assay stability.

### 3.4. Quality Assurance/Quality Control

The calibration curve for nitrate or nitrate determination were established under the different methodologies used. Linear range, sensitivity, precision detection LODs and LOQs are shown in Table 2. The obtained values indicate that this procedure provided adequate linearity in the working concentration interval 0.04–2.5 mg L^−1^ and 1.61–30 mg L^−1^ for the nitrite and nitrate, respectively (Figure S4). The LOD was calculated as 3·s/sensitivity, where s is the blank standard deviation [45], being 0.01 and 0.5 mg L^−1^ for nitrite and nitrate, respectively. The LOQ was calculated as 10 Sa/b are also listed in Table 2. Inter day and intraday relative standard deviation (%RSD) was calculated using sensors synthesized in the same batch. Using the solution method, intraday %RSD were 0.12 and 0.2, for nitrite and nitrate, respectively, while they were 0.4 and 0.7 using the sensor membrane. These results indicated satisfactory precision. In addition, a batch-to-batch precision study was performed. For this aim, the responses of the three sensors prepared in three different batches under identical conditions were obtained. The batch-to-batch interday %RSD values were 1.6 and 6.2 for nitrite and nitrate, respectively. The low %RSD values obtained gave evidence that the proposed sensors were precise for their practical application.

The analytical responses of mixtures of nitrite/nitrate were also evaluated. In order to determine both analytes, the response of the nitrite was measured first using one aliquot of the sample. A second aliquot was required to determine nitrate. When both analytes were determined in the same sample aliquot, no interference of the ZnNPs was detected in the nitrite response, and the responses of nitrite and nitrate (as nitrite) were additive.

On the other hand, the proposed assay allowed determining the concentration quantitatively by measuring the RGB components (Table 2) or semi-quantitatively by visual observation comparing the color solution with a color comparison chart. Similar responses were achieved for the single assay and for the microplate assay.

### 3.5. Storage Conditions, Stability, and Reusability

The sensor stability as a function of time and the environmental conditions was tested. Sensors kept at room temperature and protected from light and air exposition, were tested in a period of 60 days. In these conditions the sensors were totally stable. When the sensors were stored at 4 °C they were stable for at least 6 months. Figure S5 shows the signal of a given concentration of ${\mathrm{NO}}_{2}^{-}$ by using prepared sensors of different ages (0 months, 4 months, and 6 months). As can be seen, the signals were similar which meant that the reagents were stable in the PDMS supports. No differences between different synthesis were observed. The RSD% corresponding to three syntheses was <10% (*n* = 9). To evaluate the reusability, the sensor was employed with different nitrite solutions. Although the response was positive in all cases, the sensitivity was lower as the number of uses increased. Those results indicated that they were a single use.

### 3.6. Application to Real Samples

The potential utility of the proposed methodology for the determination of nitrate or nitrite in real samples (different types of real waters and liquid government from the chard and spinach canned and water from boiled vegetables) have been tested. The multiplate format was used for different water samples (drinking tap and river). As the samples contained particulate matter, the effect of filtering the samples on the nitrite or nitrate response was studied. For this purpose, aliquots of samples were first filtered and compared with non-filtered solutions. The results obtained indicated that the sample filtration did not affect the results. According to these results, samples were filtered in case of the existence of particles. In Table 3 are shown the concentrations obtained for water samples. Relatively high concentration of ${\mathrm{NO}}_{3}^{-}$ was obtained for the irrigation water compared with the other samples. These results were in concordance with those obtained by using a comparative method. Concerning the amount of ${\mathrm{NO}}_{2}^{-}$ obtained in these samples, the found concentration was below the LODs.

Regarding the liquid of the canned and the boiled water obtained from vegetables, two different samples of two different green leaves vegetables were analyzed. The samples were diluted in order to be analyzed. The concentrations of nitrate found in the samples are shown in Table 3. The accuracy was evaluated by fortifying the samples with different nitrate concentrations. For all the concentrations studied, the recoveries were about 100% with relative errors lower than 10%. Based on these results we could conclude that no matrix effect was observed in these samples and the concentration could be directly determined by using external calibration. Validation of this methodology was carried out by comparing the results with those obtained using an ISE electrode and UV spectrophotometric methods [20]. For a level of significance of 95%, there were not statistically differences between the results of both methods. The found concentrations in the liquids from canned vegetables and boiled fresh vegetables were lower than those stablished in the CE legislation [6] that establishes a maximum of 2000 mg of nitrate per kg of vegetable. However, these results indicated that the consumption of the liquid from canned vegetables or from the boiled water from fresh vegetables can result in being a high source of nitrates in the diet. Regarding the ${\mathrm{NO}}_{2}^{-}$ concentration, none of the samples analysed presented concentrations above the LODs of the method.

Drinking and tap water were also analyzed using the multiplate format. The results are shown in Figure 7 and in Table 3. The concentration of nitrite was lower than the limit of detection in all analyzed samples. Low concentrations were found in drinking and tap waters. A dilution of the sample was required for samples M1 (lake water) and M65 (river water collected after depuration station). The concentrations of ${\mathrm{NO}}_{2}^{-}$ found in these samples were 0.05 and 0.4 mg L^−1^, respectively.

### 3.7. Comparison with Other Methods

To illustrate the advantages of the developed approach, a comparative study of the proposed method with other previously proposed has been performed (Table 4). Most of the methodologies proposed in the literature use Griess reagents in solution. The use of PDMS membranes enhances the reagent stability, avoids manipulation of reagents, and improves the dosage. Therefore, method sustainability is improved.

The methods proposed in the bibliography that use Zn as reducer agent employ large amounts to carry out the reaction, ranging from column (8 cm filled with Zn granules (0.15–0.4 mm) to suspensions of Zn dust (<10 µm). Considering this, we propose the use of ZnNPs (particles size around 100 nm hydrodynamic size). The use of dispersed ZnNPs required a lower amount (0.12 mg/sample) than non-dispersed solutions or non-nanoparticles suspensions, thus improving the environmental performance. The LODs and dynamic ranges reached by the proposed methodology are similar to other methods and this is properly adjusted to sample concentrations.

In general, this approach is more sustainable, with lower values of carbon footprint and waste generated. From the economical point of view, it required small amounts of reagents, little personal time, and not much instrumentation (a smartphone can be used as the analytical instrument). Compared with reference procedures, such as ionic liquid chromatography (IC), similar LODs were reached, however IC required more time, reagents, and instrumentation. Although LODs reached by ISE electrode are lower than those achieved with the proposed method, analysis time is reduced since no sample pretreatment is necessary. The Griess method has been traditionally applied to ${\mathrm{NO}}_{3}^{-}$ by using Cd as reducing agent which is highly toxic. Moreover, the approach proposed in this paper allows processing 96 samples simultaneously using RGB coordinates as analytical signal.

## 4. Conclusions

In this work a new approach is proposed for stabilization and dosage of Griess reagent using PDMS membranes with the reagent embedded. The influence of the TEOS and IL (OMIM PF_6_) on the reagent diffusion are studied. The characterization of the membranes was performed. The PDMS membranes allow to stabilize the reagents for more than six months. Therefore, the use of ZnNPs as reductor of ${\mathrm{NO}}_{3}^{-}$ to ${\mathrm{NO}}_{3}^{-}$ was studied. Based on these results a quick assay for quantitative determination of nitrate or nitrite in real samples was developed. Nitrite was directly determined by using the Griess reagent membranes while nitrate required a reduction step with ZnNPs. The samples were directly processed, and any sample treatment was required. Good precision (RSD <8%) and detection limit of 0.01 and 0.5 mgL^−1^ for ${\mathrm{NO}}_{2}^{-}$ and ${\mathrm{NO}}_{3}^{-}$, respectively, were achieved. The approach was satisfactorily applied to the determination of ${\mathrm{NO}}_{2}^{-}$ and ${\mathrm{NO}}_{3}^{-}$ in drinking waters, irrigation and river waters, and waters from canned and fresh vegetables. The advantages of the proposed method with respect to other reported in the literature are related to the portability, low cost, short analysis time, which contribute to be a point of need (PON) method. This approach can be considered as a green and sustainable method which can be used for in situ analysis of waters from different sources (environmental waters and food waters). This procedure has been transferred to a 96 well of a multiplate format allowing the analysis of 96 samples simultaneously, using RGB as analytical signal and smartphone as analytical instrument.

## Figures and Tables

**Table 1:** Concentrations limits of nitrate or nitrite established by different regulations.

Sample	$\mathrm{Limit}\;NO_{3}^{-}$	$\mathrm{Limit}\;NO_{2}^{-}$	Regulation
**Drinking water**	50 mg L^−1^	0.5 mgL^−1^	Council Directive 98/83/EC of 3 November 1998 EPA—United States Environmental Protection Agency WHO—World Health Organization
**Groundwater**	50 mg L^−1^	-	Directive 2006/118/EC of the European Parliament and of the Council of 12 December 2006
**Sewage treatment plant**	25 mg L^−1^	-	Spanish regulation, Royal Decree 1620/2007 of 7 December
**Fresh spinach**	2500–3000 mg kg^−1^	-	Commission Regulation (EC) N^o^ 1881/2006 of 19 December 2006
**Preserved, deep-frozen, or frozen spinach**	2000 mg kg^−1^	-	Commission Regulation (EC) No 1881/2006 of 19 December 2006

**Table 2:** Figures of merites for nitrite and nitrate. (a) All the reagents in solution; (b) by using the reagents entrapped in PDMS composite and ZnNPs dispersed; (c) by using the reagents entrapped in PDMS composite and ZnNPs retained in nylon.

	Linearity (y = a + bx) (mg L^−1^)				Precision RSD (%)		LOD
	a ± s_a_	b ± s_b_	R^2^	Linear Interval (mg·L^−1^)	Intraday (*n* = 3)	Interday (*n* = 3)	(mg·L^−1^)
NITRITE							
^(a)^ Solution	0.007 ± 0.003	0.661 ± 0.004	0.99	0.02–1.5	0.12	0.9	0.005
^(b)^ Sensor	0.0035 ± 0.009	0.551 ± 0.007	0.99	0.04–2.7	0.4	1.6	0.01
^(b)^ RGB (green)	0.062 ± 0.013	0.550 ± 0.004	0.99	0.09–1.3	1.1	7.2	0.03
^(b)^ RGB (green) multiplate	0.079 ± 0.013	0.56 ± 0.08	0.99	0.02–2.7	1.3	4.3	0.01
NITRATE							
^(a)^ Solution	0.028 ± 0.013	0.056 ± 0.001	0.99	0.3–30	0.2	4.3	0.1
^(b)^ Sensor	0.034 ± 0.007	0.0409 ± 0.005	0.99	1.6–30	0.7	5.8	0.5
^(c)^ Sensor	0.031 ± 0.008	0.038 ± 0.0004	0.99	1.6–30	0.2	6.2	0.5
^(b)^ RGB (green)	0.114 ± 0.013	0.052 ± 0.003	0.99	2.8–25	1.1	7.2	0.8
^(b)^ RGB (green) Microplate	0.04 ± 0.013	0.0532 ± 0.004	0.97	0.7–20	0.8	7.0	0.2

**Table 3:** Found concentrations of ${\mathrm{NO}}_{3}^{-}$ in different real samples (irrigation water samples, water from canned and boiled fresh green vegetables, river, drinking and tap water). Recoveries corresponding to nitrate (spiled samples ${\mathrm{NO}}_{3}^{-}$ 5 mg L^−1^) fortified samples. Comparative method ^(a)^ UV molecular spectrophotometry, ^(b)^ ${\mathrm{NO}}_{3}^{-}$ ISE electrode. (*) 96 multiplate assay.

Samples		$\mathrm{Found}\;\mathrm{Concentration}\;NO_{3}^{-}\;(\mathrm{mg}\;L{-}^{1})\;(n=3)$		Recovery (%)
		Proposed Method	Comparative Method	
**Irrigation water**	Chanel	69.2 ± 0.9	67.13 ± 0.2 ^(a)^	97.2 ± 0.2
	Well	21.42 ± 0.05	21.6 ± 0.1 ^(a)^	100.5 ± 0.5
	Well	2.79 ± 0.01	----	----
**Canned vegetables**	Chard	1103 ± 60	1210 ± 90 ^(b)^	103.9 ± 0.4
	Spinach	810 ± 40	-	107.3 ± 0.2
**Fresh vegetables**	Chard	1600 ± 100	1700 ± 70 ^(b)^	93.0 ± 0.4
	Spinach	990 ± 40	980 ± 70 ^(b)^	104.0 ± 0.5
Drinking water (*) Tap water (*)	M84 M85 M86 M87 M88 M89 M90 M81 M91	10.6 2.8 <LOD 1.8 2.0 2.0 2.2 7.0 6.0		
River water (*) Lake water (*)	M65 M1	74 204		

**Table 4:** Main analytical properties of different procedures described in the literature for ${\mathrm{NO}}_{2}^{-}/{\mathrm{NO}}_{3}^{-}$ ^-^determination. ^(a)^ ${\mathrm{NO}}_{2}^{-},$ ^(b)^ ${\mathrm{NO}}_{3}^{-}.$ (*) the greater the number of asterisks, the greater the parameter.

Analyte/ Sample	Procedure	Evaluation					Reference
		Analytical Parameters		Green Points		Economical Points	
		Analytical Time/ Robustez	Figures of Merits LODs/ Dynamic Range	Footprint Kg CO_2_/100 Samples	Waste	Reagent Consumption Personal Time Instrument	
${\mathrm{NO}}_{3}^{-}$ (water)	Nitrate Reduction— column Zn granules (0.15–0.40 mm) 8 cm 3 min. Derivatization Griess—solution Analytical signal— Absorbance	FIA in lab or in situ (40 samples/h)	0.006 mg L^−1^ 0.04–0.3 mg L^−1^	0.25	High amount of waste (Continuous flow)	Reagents: *** Personal: ** Instrument: ***	[24]
${\mathrm{NO}}_{2}^{-}/{\mathrm{NO}}_{3}^{-\to }$ (water and food samples)	Nitrate Reduction—Zn powder (0.1 g/sample)-5 min volume 100 mL Derivatization Griess—10 min Analytical signal—Absorbance	Batch	3 to 5 mg Kg^−1^	5.99	High volumes used	Reagents: *** Personal: *** Instrument: **	[40]
${\mathrm{NO}}_{3}^{-}$ (water)	Nitrate Reduction—Zn powder (150 µm) (25 mg/sample) 10 min Derivatization Griess—solution Anaytical signal—Absorbance	Batch	0.5 mg L^−1^ 0.5–45 mg L^−1^	0.23	10 mL sample/1 min reagent	Reagents: ** Personal: *** Instrument: **	[41]
^(a)^${\mathrm{NO}}_{2}^{-}$^(b)^${\mathrm{NO}}_{3}^{-\to }$ (Synthetic, tap, pond, and mineral water)	Inkjet printing with AKD Zn suspension prepared by mixing 500 mg of Zn dust (<10 µm); 1 mg/sample (75 s) Derivatization Griess—µPAD paper support (3–7 min) Analytical signal—scanned imagens processed	In situ Microfluidic Sensor Stable for 30 days Stored in vacuum at ≤−20 °C)	^(a)^ 0.04/^(b)^ 1.2 mg L^−1^ 0.5–6.9 mg L^−1 (a)^ 3.1–62 mg L^−1 (b)^	9.45 In the fabrication of the sensor/scanner	Low consumption of reagents (µL)	Reagents: * Personal: * Instrument: **	[30]
^(a)^${\mathrm{NO}}_{2}^{-}$/^(b)^${\mathrm{NO}}_{3}^{-}$	Nitrate Reduction—Dispersion of ZnNPs (0.12 mg/sample) 3 min Derivatization Griess—5 min, solution Analytical signal—processed imagens (RGB) or Absorbance	In situ Reagents supported in PDMS Stable for more than 6 moths	^(a)^ 0.01/^(b)^ 0.5 mg L^−1^ 0.04–2.5 mg L^−1 (a)^ 1.6–30 mg L^−1 (b)^	0.028	Low consumption of reagents (µL)	Reagents: * Personal: * Instrument: *	Proposed method
Reference methods							
${\mathrm{NO}}_{3}^{-}$	Electrode Nitrate 10 mL sample 10 mL of buffer (Al_3_SO_4_)_3_ 17 mg; Ag_2_SO_4_ 34.3 mg; H_3_BO_4_ 18.6 mg, H_2_SO_3_H 25.4 mg/per sample) Analytical signal—Electrochemical	In situ in the lab Interferences: Cl^−^, CO_3_^−2^, NO_2_^−^, CN^−^, S^2−^, Br^−^, I^−^, ClO_3_^−^, ClO_4_^−^	0.6 mg L^−1^ 0.6–620 mg L^−1^	1.8 × 10^−4^	High volumes	Reagent: ** Time/sample: 3 min Instrumentation: ***	[32]
${\mathrm{NO}}_{3}^{-}$	Absorbance measurement at 220 nm and 275 nm Analytical signal—Absorbance	In situ (probes) In the lab (batch or FIA) Interferences: soaps, NO_2_^−^, Cr^6+^	0.1 mg L^−1^ 0.3 a 30 mg L^−1^	0.025		Reagent: Time/sample: 1 min Instrumentation: ***	[32]

## Data Availability

The data presented in this study are available on request from the corresponding author.

## Supplementary Materials

The following supporting information can be downloaded at: https://www.mdpi.com/article/10.3390/polym14030464/s1. Figure S1: FT-IR spectra of PDMS-IL (blue), PDMS-IL-SA-NEDD (red), SA (grey) and NEDD (black), Figure S2: EDX spectra a) PDMS, b) PDMS-IL, c) PDMS-IL-SA-NED, Figure S3: Imagens of optic microscopy for A) solution of SDS:CATB (30:70) by and B) ZnNPs dispersed on solution of SDS:CATB (30:70), Figure S4: Calibration graphs corresponding to the reagents in solution for ${\mathrm{NO}}_{2}^{-}$ (1) and ${\mathrm{NO}}_{3}^{-}$ (3) and for the sensor for ${\mathrm{NO}}_{2}^{-}$ (2) and ${\mathrm{NO}}_{3}^{-}$ (4), Figure S5: Absorbance vs. time for different age sensors (0, 4 and 6 months).
