# Supplementary material for: Improving Sustainability of the Griess Reaction by Reagent Stabilization on PDMS Membranes and ZnNPs as Reductor of Nitrates: Application to Different Water Samples
Source: Polymers (Basel). 2022 Jan 24;14(3):464. doi: 10.3390/polym14030464 (PMC8839667; doi:10.3390/polym14030464)
Supplement: Supplementary file 1 [file polymers-14-00464-s001.zip › polymers-1537599-supplementary.pdf]

polymers

Supplementary Materials

Improving Sustainability of the Griess Reaction by Reagent Stabilization on PDMS Membranes and ZnNPs as Reductor of Nitrates: Application to Different Water Samples

Lusine Hakobyan, Belén Monforte-Gómez, Yolanda Moliner-Martínez, Carmen Molins-Legua * and Pilar Campíns-Falcó *

MINTOTA Research Group, Departament de Química Analítica, Facultat de Química, Universitat de València, Dr. Moliner 50, 46100 Valencia, Spain; lusine.hakobyan@uv.es (L.H.); mongobe@alumni.uv.es (B.M.-G.); yolanda.moliner@uv.es (Y.M.-M.)

* Correspondence: cmolins@uv.es (C.M.-L.); pilar.campins@uv.es (P.C.-F.)

Table of Contents

- **Figure S1.** FT-IR spectra of PDMS-IL (blue), PDMS-IL-SA-NEDD (red), SA (grey) and NEDD (black)
- **Figure S2**. EDX spectra a) PDMS, b) PDMS-IL, c) PDMS-IL-SA-NED
- **Figure S3.** Imagens of optic microscopy for A) solution of SDS:CATB (30:70) by and B) ZnNPs dispersed on solution of SDS:CATB (30:70).
- **Figure S4.** Calibration graphs corresponding to the reagents in solution for NO_2^- (1) and NO_3^- (3) and for the sensor for NO_2^- (2) and NO_3^- (4).
- **Figure S5**. Absorbance vs time for different age sensors (0, 4 and 6 months).

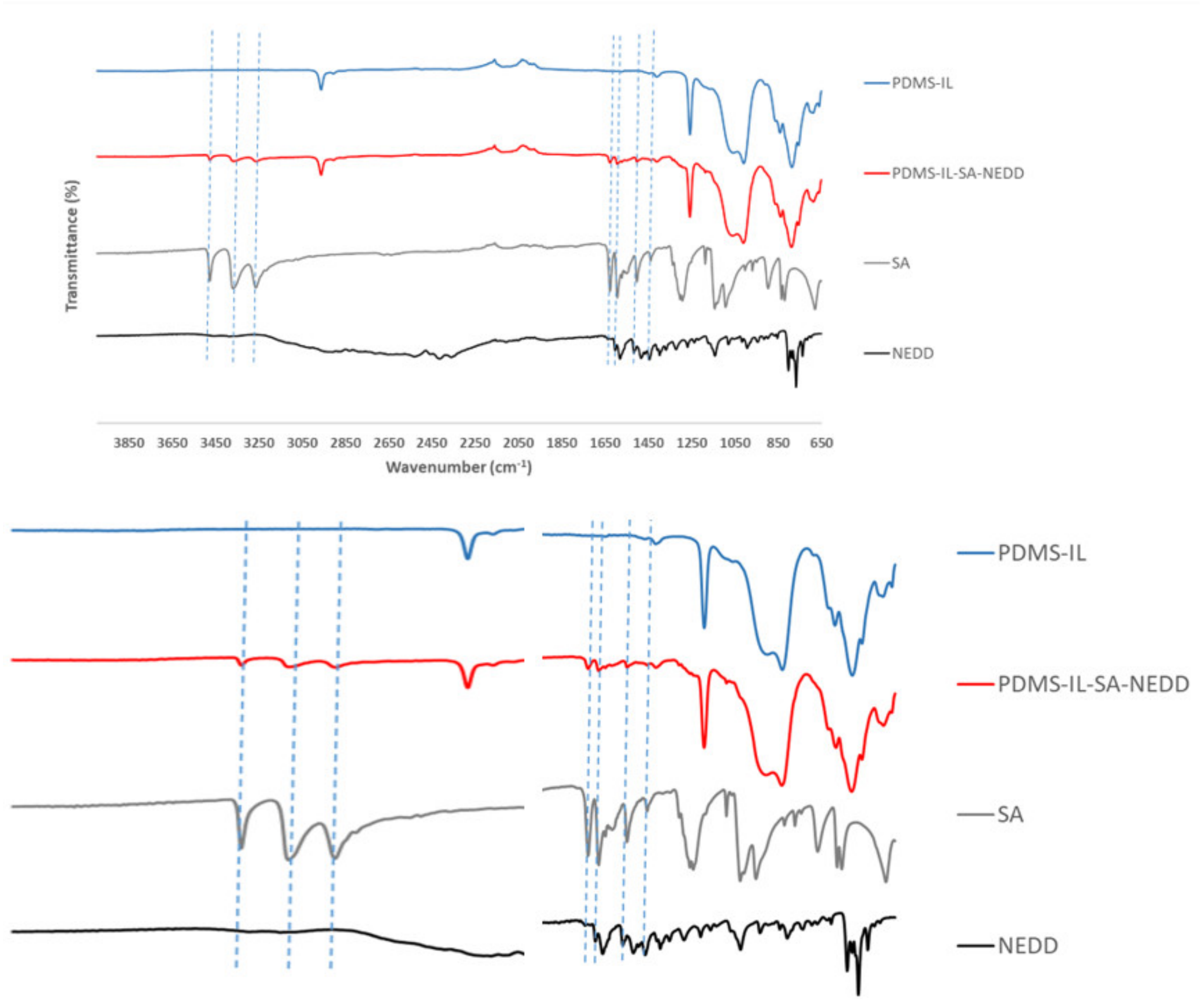

Figure S1. FT-IR spectra of PDMS-IL (blue), PDMS-IL-SA-NEDD (red), SA (grey) and NEDD (black).

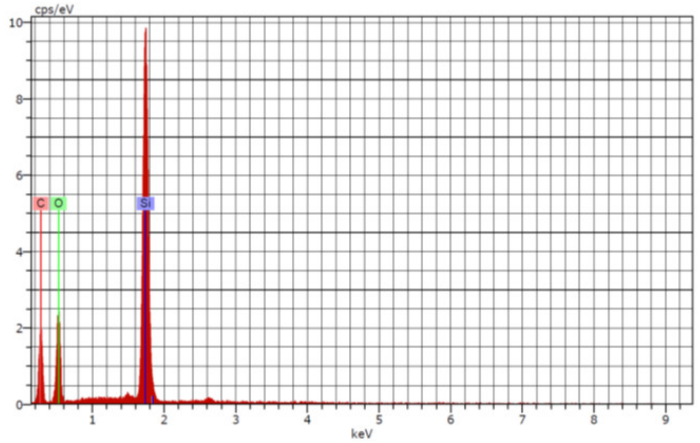

(**a**)

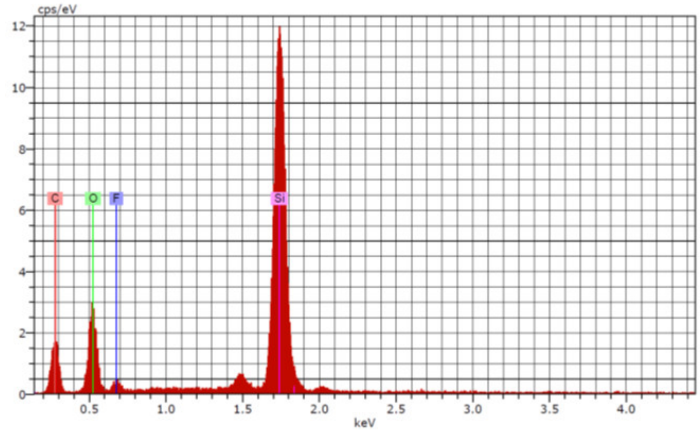

(**b**)

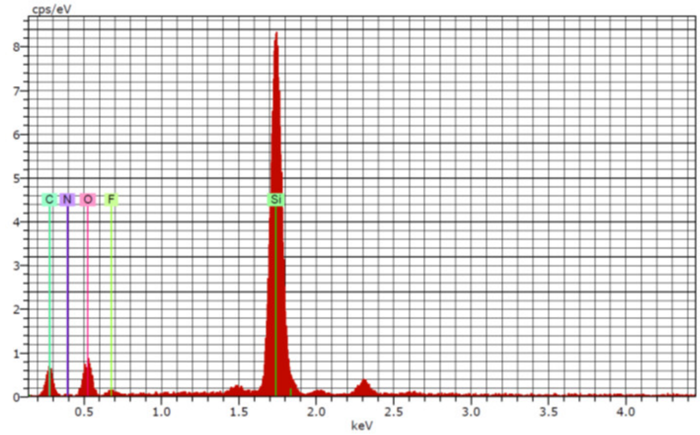

(**c**)

Figure S2. EDX spectra (**a**) PDMS, (**b**) PDMS-IL, (**c**) PDMS-IL-SA-NED.

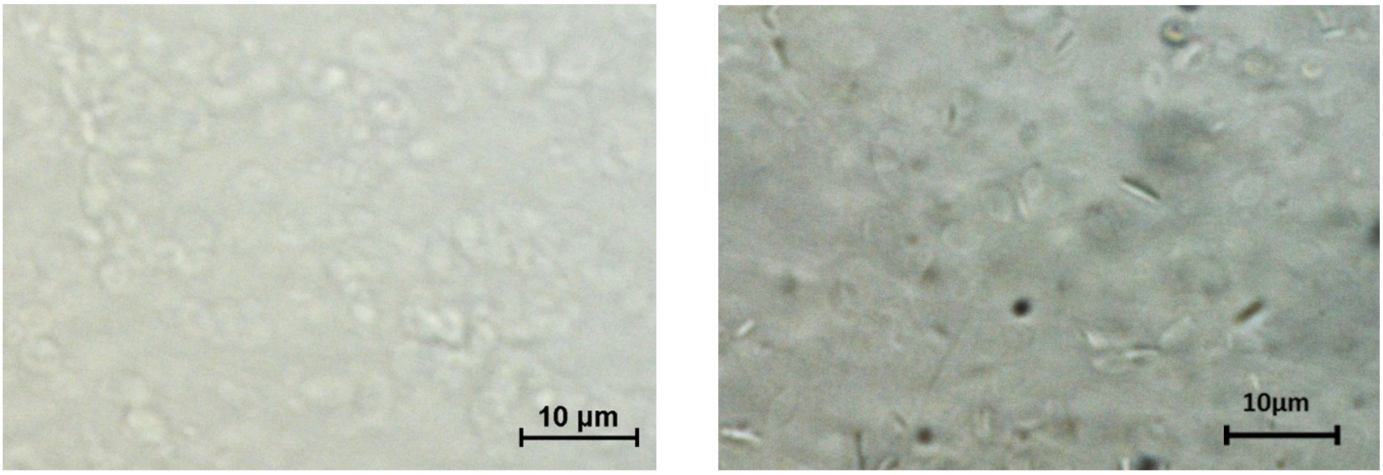

(A) **(B)**

Figure S3. Imagens of optic microscopy for (**A**) solution of SDS:CATB (30:70) by and (**B**) ZnNPs dispersed on solution of SDS:CATB (30:70).

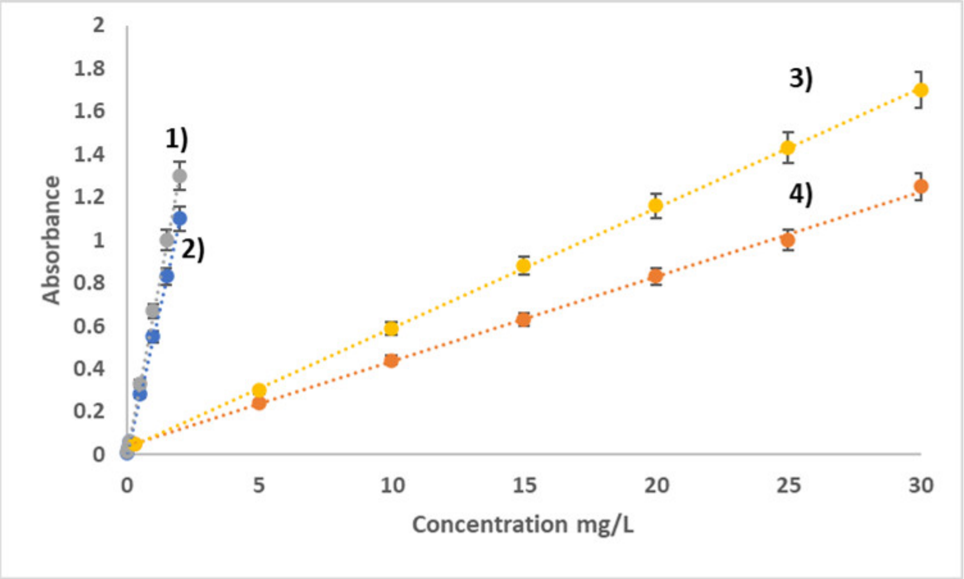

Figure S4. Calibration graphs corresponding to the reagents in solution for NO_2^- (**1**) and NO_3^- (**3**) and for the sensor for NO_2^- (**2**) and NO_3^- (**4**).

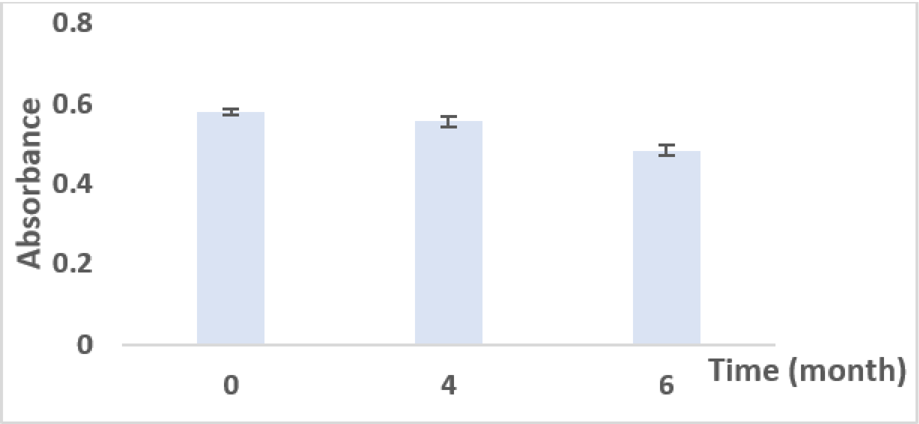

Figure S5. Absorbance vs time for different age sensors (0, 4 and 6 months).
